# Morphological analysis of posterior-medial intertrochanteric fracture patterns using fracture-mapping technique

**DOI:** 10.3389/fbioe.2023.1275204

**Published:** 2023-11-09

**Authors:** Hanru Ren, Xu Zhang, Yakun Liang, Chengqing Yi, Dejian Li

**Affiliations:** ^1^ Department of Orthopedics, Shanghai Pudong Hospital, Fudan University Pudong Medical Center, Shanghai, China; ^2^ Shanghai Institute of Precision Medicine, Shanghai Ninth People’s Hospital, Shanghai Jiao Tong University School of Medicine, Shanghai, China; ^3^ Research Institute of Digital and Intelligent Orthopedics, Fudan University Pudong Medical Center, Shanghai, China

**Keywords:** fracture-mapping technique, posterior-medial fragment, intertrochanteric fracture, new classification, 3D-CT

## Abstract

**Introduction:** The purpose of this study was to analyze the fracture patterns of different posterior-medial wall types of intertrochanteric fractures by 3-D fracture-mapping technique and to further assess their clinical utility.

**Methods:** In a retrospective analysis of interochanteric fractures treated in a large trauma center, fractures were classified into predesigned groups based on 3D-CT imaging techniques, and a 3-D template of the intertrochanteric region was graphically superimposed on the fracture line. Fracture characteristics were then summarized based on fracture-mapping. Finally, radiographic parameters, function, and range of motion were recorded in different fracture classification states.

**Results:** A total of 348 intertrochanteric fractures were included. There were 111 patients (31.9%) in the posterolateral + posteromedial + medial group, with the most severe fracture displacement (typically characterized by fragmentation of the posteromedial wall into three isolated fragments). There were 102 cases (29.3%) in the posterolateral + posteromedial + simple medial group, and the most common fracture feature was a complete fragment posteromedially. A total of 81 cases (23.3%) were classified into the posterolateral + medial group, with the medial fracture line extending the anterior fracture line but leaving the lesser trochanter intact. In the isolated medial group of 33 cases (9.5%), the fracture type was similar to type IV, but the integrity of the greater trochanter was ensured. In the posteromedial + medial group of 12 cases (3.4%), the fracture was characterized by an interruption when the fracture line of the anterolateral wall extended to the posteromedial wall, often resulting in a complete isolated fragment posteromedially and medially. There were nine patients (2.6%) in the isolated posterolateral group. In addition, we found significantly different radiographic scores and range of motion scores between groups.

**Discussion:** This morphometric study helps us to further characterize posterior-medial fracture patterns of intertrochanteric fractures, which may be closely related to different clinical outcomes. Further studies are needed to verify the reliability of this classification scheme in clinical application.

## Background

Intertrochanteric fracture of the femur is a common type of fracture in older individuals (Socci et al., 2017). If the poor quality of fracture reduction, premature weight-bearing exercise after fracture surgery may lead to serious complications such as fracture re-displacement and cut out, but long-term bed-rest immobilization may also increase the incidence of postoperative complications (Frei et al., 2012; Hua et al., 2014; Tan et al., 2016); hence, femoral intertrochanteric fracture is still considered as an “unsolved fracture” type at present (Haidukewych, 2009; Haidukewych, 2010).

**FIGURE 1:**
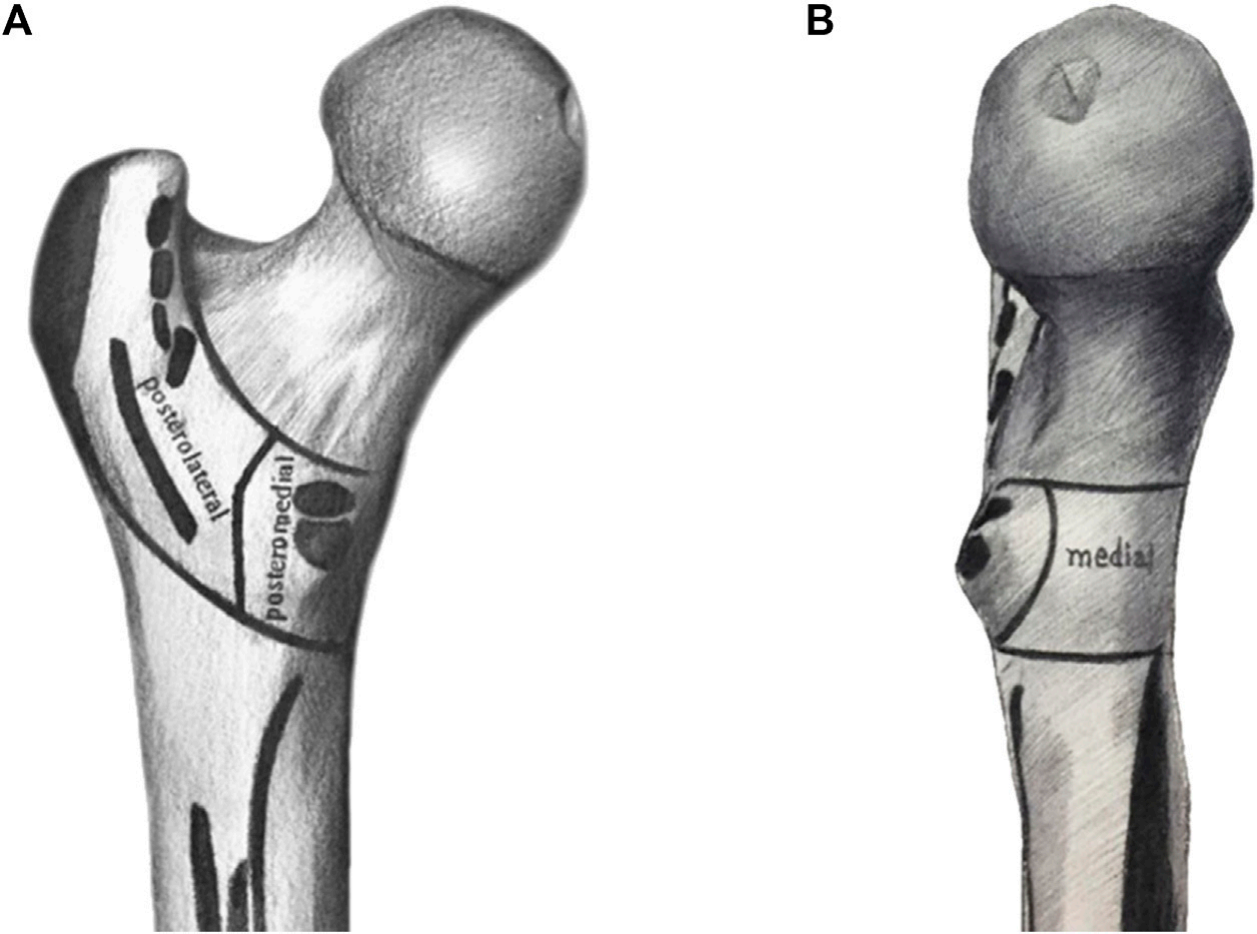
The posterior and medial structures between trochanters is divided into posterolateral, posteromedial, and medial structures. The posterolateral refers to the area near the greater trochanter, posteromedial refers to the area covering the entire smaller trochanter, and medial refers to the area of medial wall. **(A)** posterior aspect; **(B)** medial aspect.

The posterior wall and medial wall are important biological structures of the femoral trochanter (Sharma et al., 2014). Previous studies have shown that the integrity of posterior-medial structure is closely related to its stability after fracture surgery and hip joint functional mobility (Ciufo et al., 2017; Sharma et al., 2017). Marmor et al. suggest that in the process of intramedullary nail treatment (Marmor et al., 2013), fractures become more and more unstable as the severity of medial cortical fragmentation increases, but previous studies often regard the whole posterior wall and medial wall as a whole, and lack further understanding of its structure and biological characteristics. However, with the wide application of CT three-dimensional reconstruction technique in treating intertrochanteric fractures and further understanding of the structures of posterior-medial wall (Futamura et al., 2016; Shoda et al., 2017), we believe that the biological characteristics of the posterolateral (the area near the greater trochanter, with the stop point near the medial edge of the quadratus femoris muscle), posteromedial (the area covering the entire small trochanter) and medial intertrochanteric (the pressure-bearing area of the femoral trochanter) parts may be different (Figure 1), and the possible postoperative complications and precautions caused by fractures in different regions may also be different (Ren et al., 2019; Ren et al., 2020). The medial side of the proximal femur is an important supportive structure. Insufficient medial support after surgery may lead to deformities such as the inward rotation of the femoral head and the disappearance of the neck-shaft angle. The posterior side of the proximal femur includes the lesser tubercle and part of the greater tubercle; they serve as attachment points for some tendons. Improper treatment after surgery could result in partial loss of hip joint mobility. With the wide application of CT three-dimensional (3-D) reconstruction techniques in treating intertrochanteric fractures coupled with further understanding of the structures of posterior-medial wall, this issue can now be explored in detail. The purpose of this study was to classify the fracture types in different posterior-medial regions through CT reconstruction, and to understand the fracture patterns between different types through fracture-mapping techniques. We also aimed to further explore the influence of different fracture regions and classification systems on postoperative function and complications of fractures.

## Methods

### Subjects

This retrospective study received ethics approval from our institution and analyzed patients diagnosed with femoral intertrochanteric fracture in the orthopedic database from January 2018 to December 2020 in a Chinese trauma center. Inclusion criteria include the following: 1) The patient was aged over 60 years; 2) CT examination confirmed patients with posterior-medial wall fractures; 3) Fresh fracture, injury to operation time <2 weeks; 4) Closed fracture. Exclusion criteria include: 1) AO-3 intertrochanteric fracture with fracture line passing through lateral cortex; 2) Combined with multiple injuries; 3) Pathological fracture; 4) Congenital dysplasia of hip; 5) Severe osteoporosis; 6) Difficulty walking due to serious internal diseases before injury (e.g., tumor, Parkinson’s disease); 7) Follow-up time is less than 1 year.

### Bone block area

**FIGURE 2:**
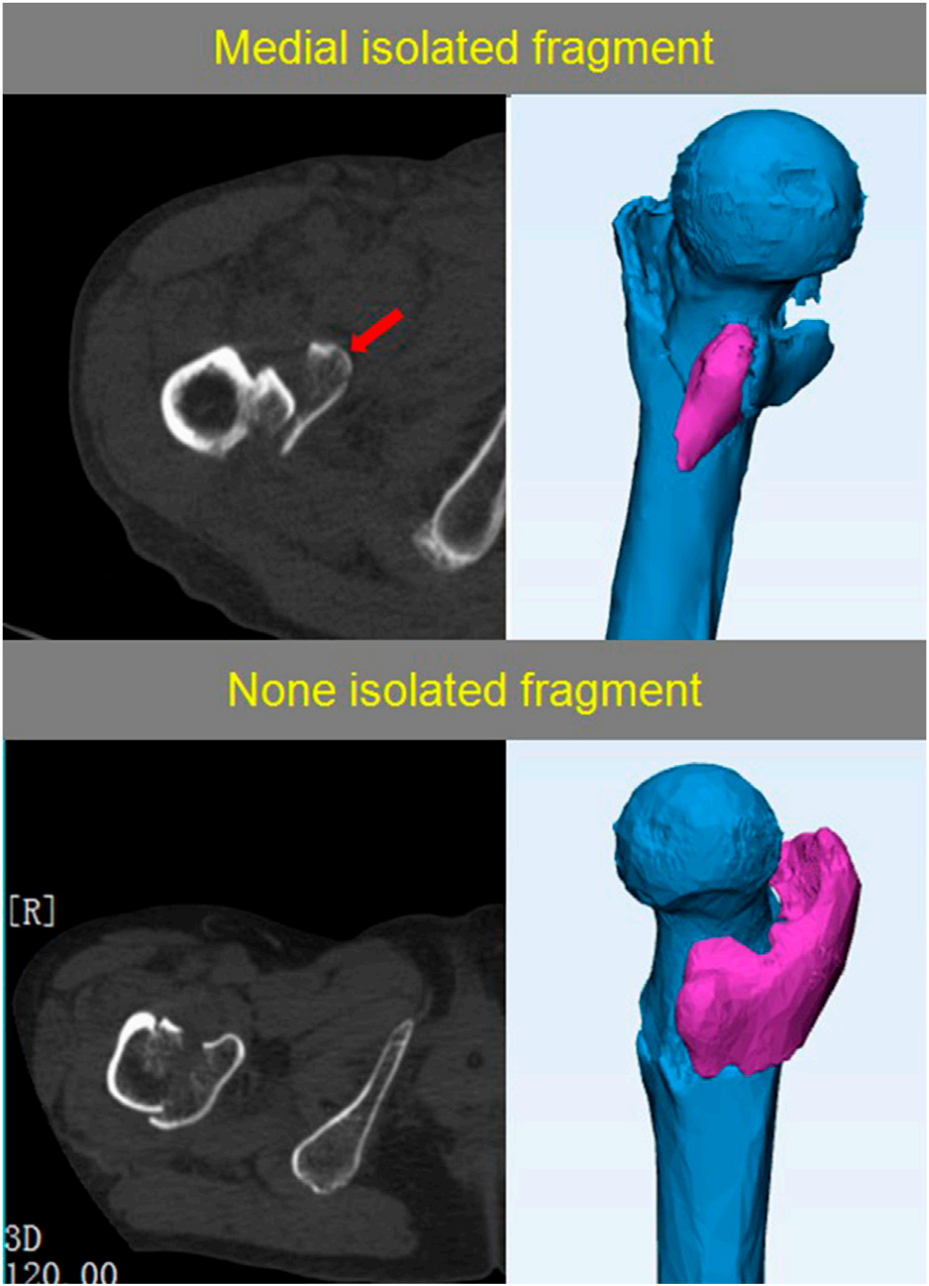
Using 2-d and 3-d CT images to determine medial isolated fragment. The red arrow shows medial isolated fragment.

According to anatomical characteristics, the posterior-medial structure of trochanters can be divided into posterolateral, posteromedial, and medial bone block areas (Figure 1), in which the medial edge of the posterolateral is located at the medial edge of the quadratus femoris muscle, and the posterior medial covers the entire trochanter area. We assume that the 99 posterolateral and posteromedial parts alone do play a secondary supporting role in force. The posterolateral part is the stop point of multiple abductor muscle groups and the posteromedial part is the stop point of adductor muscle groups, and this part of the injury is related to postoperative functional recovery of hip joint. The medial part plays an important supporting role and is related to complications such as postoperative re-displacement. Therefore, according to our regional grouping, we divided the posterior and medial fracture regions into seven groups: isolated posterolateral group, isolated posteromedial group, isolated medial group, posterolateral + posterior medial group, posterior medial + medial group, posterolateral + medial group, and posterolateral + posterior medial + medial group (Figure 2). Additionally, due to the very high probability of intertrochanteric fracture accompanied by medial wall injury, we also divided the medial wall fracture group into simple medial group and isolated fragment medial groups, because we believe that isolated medial fragment may be an important reason for the lack of support after medial wall surgery.

All fracture regions were grouped through two-dimensional (2-D) CT images and 3-D reconstructed images in PACS (Picture Archiving Communication System) and independently reviewed by three orthopedic doctors experienced in treating femoral intertrochanteric fractures. Conflicting viewpoints were resolved by group discussion.

### Fracture mapping

**FIGURE 3:**
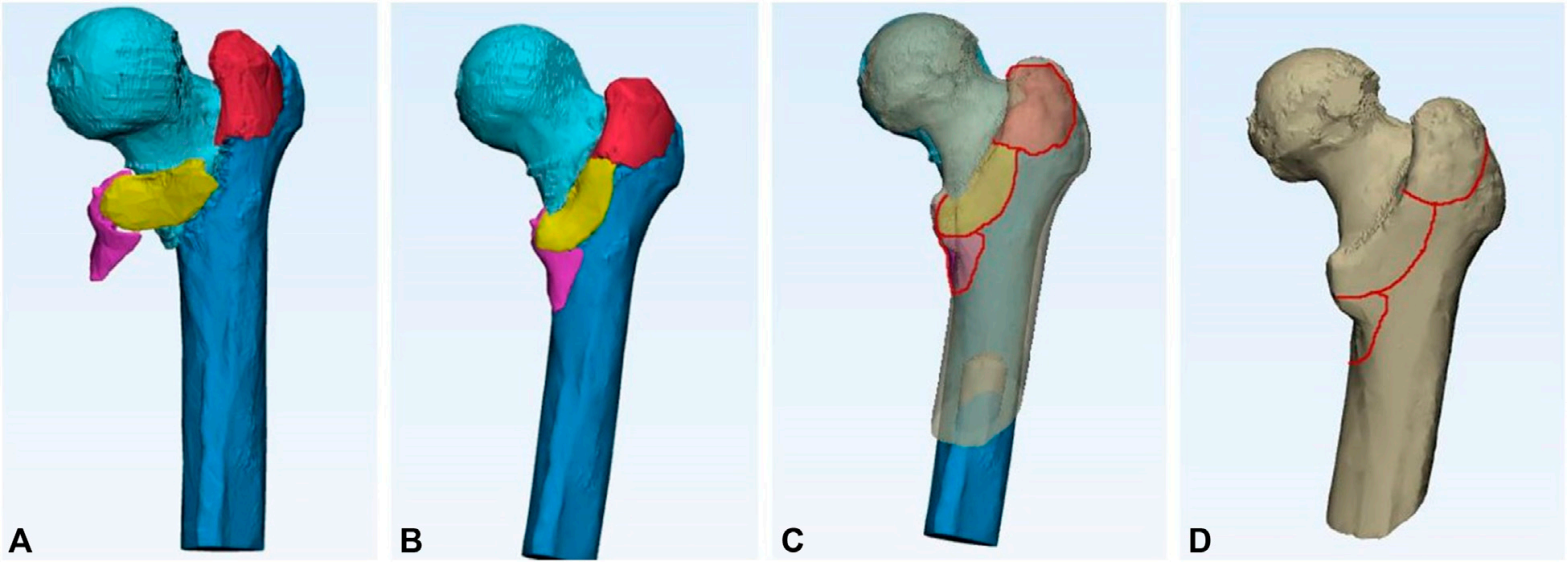
Three-dimensional fracture-mapping technique is used to prove the spatial morphology of femoral intertrochanteric fracture. CT data were used for reconstruction and virtually reduced fractures **(A,B)**. Then, if necessary, other processes were performed to rotate, normalize, and flip the image to best match the 3-dimensional template of the femoral trochanter **(C)**. Smooth curves are directly drawn on the surface of the 3-D model to represent fracture lines **(D)**.

Three-dimensional fracture-mapping technique was used to prove spatial morphology of femoral intertrochanteric fractures (Xie et al., 2017). CT data were used for reconstruction and virtually reduced fractures. Rotation, normalization, and flipping of the image were performed as needed to best match the 3-D template of the femoral trochanter (3-matic software; Materialise). Reference was made to landmarks, including bone contours of medial and lateral trochanters, greater trochanter, lesser trochanter, intertrochanteric ridge, pubic line gluteus trochanter, femoral neck, femoral head, and femoral shaft for alignment and standardization. Smooth curves were directly drawn on the surface of the 3-D model to represent fracture lines, and all fracture lines were overlapped onto the 3-dimensional model to produce a spatial fracture map. Then, each graph was combined for each fracture type to generate overall fracture-mapping (Figure 3).

### Radiography parameters and function

The follow-up time of the patients in this study was 1 week and 12 months after surgery. The measurement of radiography parameters includes the change of femoral neck–shaft angle (FNSA), and the patient’s hip Harris score (HHS) is used to evaluate the postoperative function of the patient. FNSA was determined by applying Hologic 1000 DXA bone densitometry analysis, which is the international standardized measure men, and the sliding distance of cephalic nail is measured using the method mentioned in our previous paper (Ren et al., 2020).In addition, the tip-apex distance (TED) in the patient 1 week after surgery was also measured, as it indicates the stability of repair in the intertrochanteric fractures.

### Range of motion

The measurement of hip joint motion range include flexion/extension, adduction/abduction and internal/external rotation. All the measurements follow the test procedure described by Norkin et al. (1995). The follow-up time of the patients in this study was 1 week and 12 months after surgery.

All statistical analyses were performed using SPSS 22.0 statistical software (SSPS, Chicago, IL). Measurement data are presented as means and standard deviations. A comparison between categorical data was performed with chi-square and Fisher’s exact tests. In all tests, a *p* value less than .05 was considered statistically significant.

## Results

**FIGURE 4:**
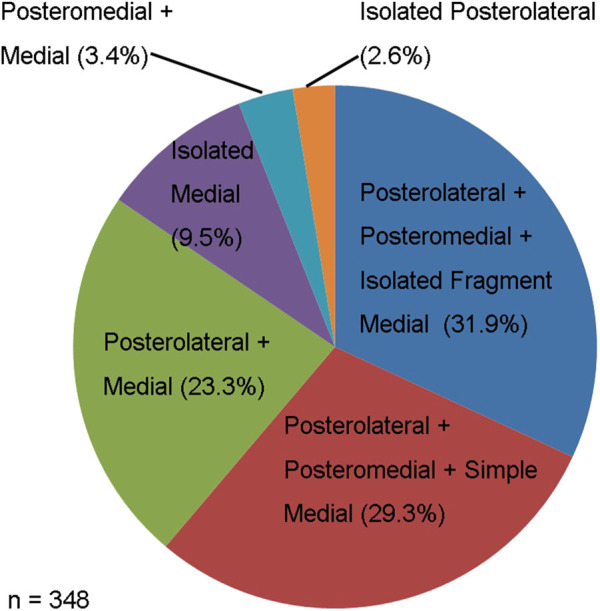
The distribution of different posterior-medial wall injury type.

A total of 144 males and 204 females (*n* = 348) with intertrochanteric fractures were included in this study, with a mean age of 73.5 years (Table 1). No patients were allocated to the isolated posteromedial group or posterior + posteromedial group. Instead, we found that almost all patients had medial wall injuries (339/348, 97.41%). Of all of the patients, 213 (61.21%) were allocated to the posterolateral + posteromedial + medial group, and we further divided them into the posterolateral + posteromedial + isolated fragment medial group and the posterolateral + posteromedial + simple medial group. Furthermore, 111 (31.9%) and 102 (29.3%) patients were in the posterolateral + posteromedial + isolated fragment medial group and posterolateral + posteromedial + simple medial group, respectively, and 81 (23.3%), 33 (9.5%), 12 (3.4%), and 9 (2.6%) in the posterolateral + medial group, the isolated medial group, the posteromedial + medial group, and isolated posterolateral group, respectively (Figure 4).

**TABLE 1:** Demographic data and baseline characteristics.

	Case(*n*)
Age (years)	73.5 ± 7.31
Gender (male/female)	144/204
Weight (kg)	62.31 ± 11.27
Length of stay (day)	6.93 ± 2.21
follow-up (month)	17.13 ± 4.33

### Posterolateral + posteromedial + isolated fragment medial group (type I)

**FIGURE 5:**
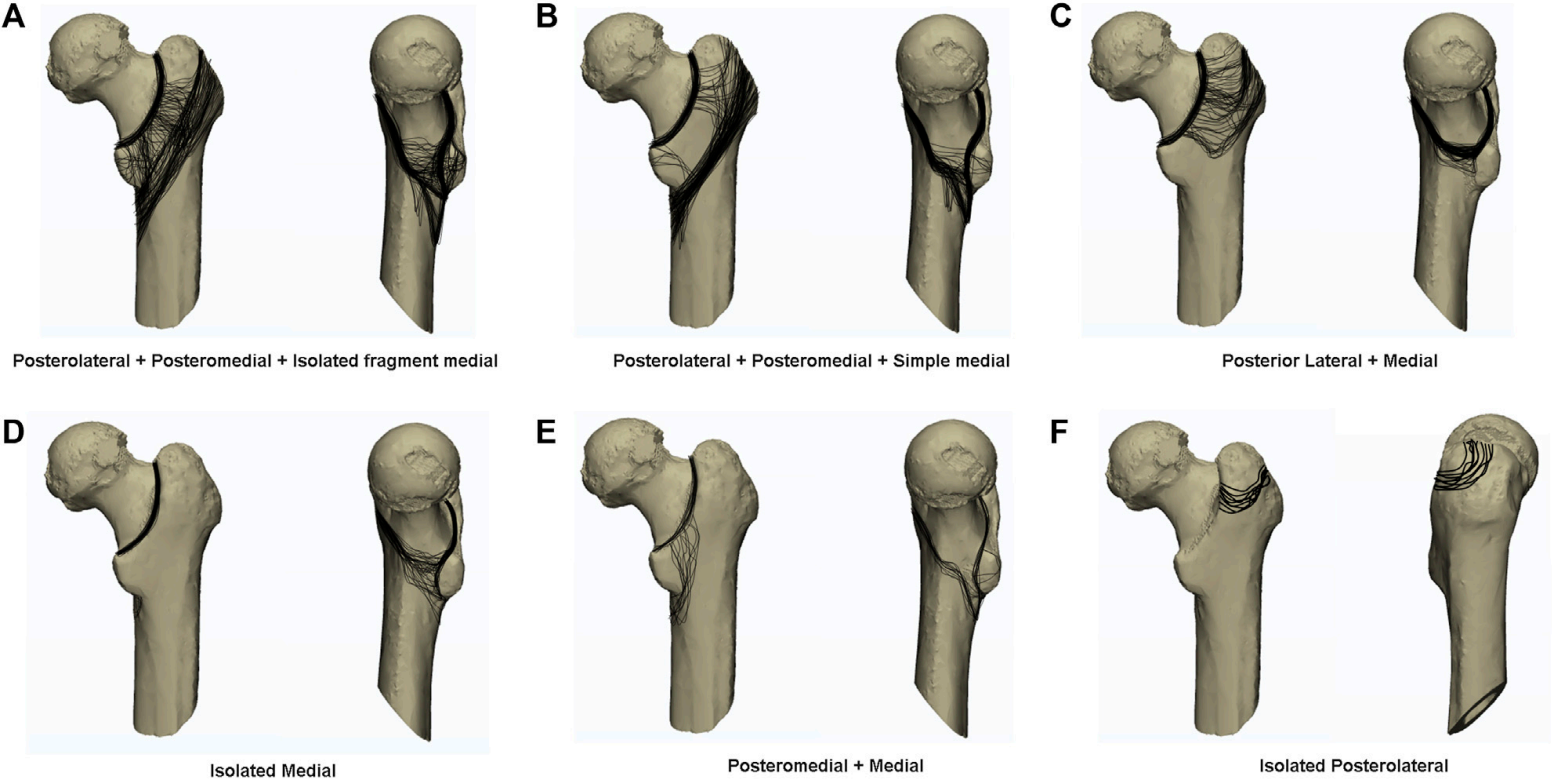
Representative views of the 3-dimensional maps of the six Intertrochanteric posterior-medial fracture types. Fracture lines are depicted in black.

**FIGURE 6:**
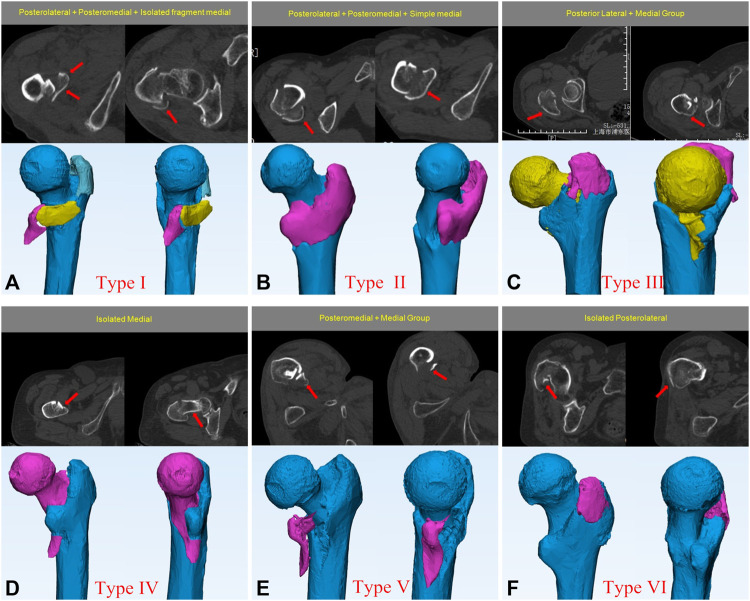
The most common fracture characteristics of different posterior-medial injurie types. Using CT images to determine fracture morphology and fragment. **(A)** Posterolateral + Posteromedial + Isolated fragment medial group. **(B)** Posterolateral + Posteromedial + Simple medial group. **(C)** Posterior Lateral + Medial group. **(D)** Isolated Medial group. **(E)** Posteromedial + Medial group. **(F)** Isolated Posterolateral group.

In total, 111 fractures were included in this group for analysis. This group of patients had the most serious fracture displacement, including 66 patients with the posteromedial wall fractured into three fragments [posterolateral fragments (greater trochanteric region), posterior-medial fragments (lesser trochanteric region), and medial fragments (medial wall)] (Figures 5A, 6A). The pattern of fractures in the other 33 patients was posteromedial separation into two fragments, which may be posterior (posteromedial + posterolateral) and medial fragments, or posteromedial + medial fragments and posterolateral fragments. There were also 12 patients whose fractures were severely displaced with extremely small fragments, and could not be systematically classified.

### Posterolateral + posteromedial + simple medial group (type II)

Among the 102 fractures in this group, the most common feature was the presence of a posteromedial intact fragment (77 patients in total) including posterolateral + posteromedial + medial involvement (Figures 5B, 6B). Moreover, 2 posterior-medial fragments (a posterolateral fragment and a medial + posteromedial intact fragment) was observed in 24 patients, with few patients found to have a posterior-medial fracture line located between the posterolateral and posteromedial sides.

### Posterior lateral + medial group (type III)

Overall, 81 fractures were analyzed. The fracture characteristics of this group were as follows: the greater trochanter fragment at the posterolateral side and the fracture fragment at the medial wall (Figures 5C, 6C). The fracture line at the medial wall was found to be an extension of the fracture line at the anterolateral wall. Moreover, the position of the fracture line at the medial wall was significantly higher than that of type I and type II (*p* < 0.01), and the smaller trochanter was bypassed, so that the smaller trochanter could remain intact. This group of fractures can sometimes form isolated medial fragments, but in this group of patients, the shape of the posteromedial small area remained intact without fracture line extension.

### Isolated medial group (type IV)

Thirty-three fractures were analyzed, and the common fracture features in this group of patients were that the fracture line of their medial wall was an extension of the fracture line of the anterolateral wall (Figures 5D, 6D), which could be seen extending to the base of the femoral neck on the posterior side, and retaining the intact femoral calcar. Of the 30 patients, only 3 patients endured a medial wall isolated fragment separated from the anterolateral wall.

### Posteromedial + medial group (type V)

We analyzed 12 fractures in this group. The fracture characteristics of this group of patients were that the fracture line interrupted when the anterolateral wall extended to the posteromedial wall, which formed a complete isolated fragment posteromedially and medially (Figures 5E, 6E). Otherwise, the posteromedial and medial separation of two isolated fragments was observed in 12 patients.

### Isolated posterolateral group (type VI)

The group exhibited 9 fractures, of which patients had a relatively typical greater trochanter fracture (Figures 5F, 6F). All patients had visible injuries to the anterior wall, and the fracture was a separate fragment at the greater trochanter. Because of the retrospective nature of the study, which collected patients undergoing surgery, conservative treatment was chosen by the majority of patients with greater trochanteric fractures such that the number of patients counted in this group may have been insufficient.

### Radiography parameters, function, and range of motion

**FIGURE 7:**
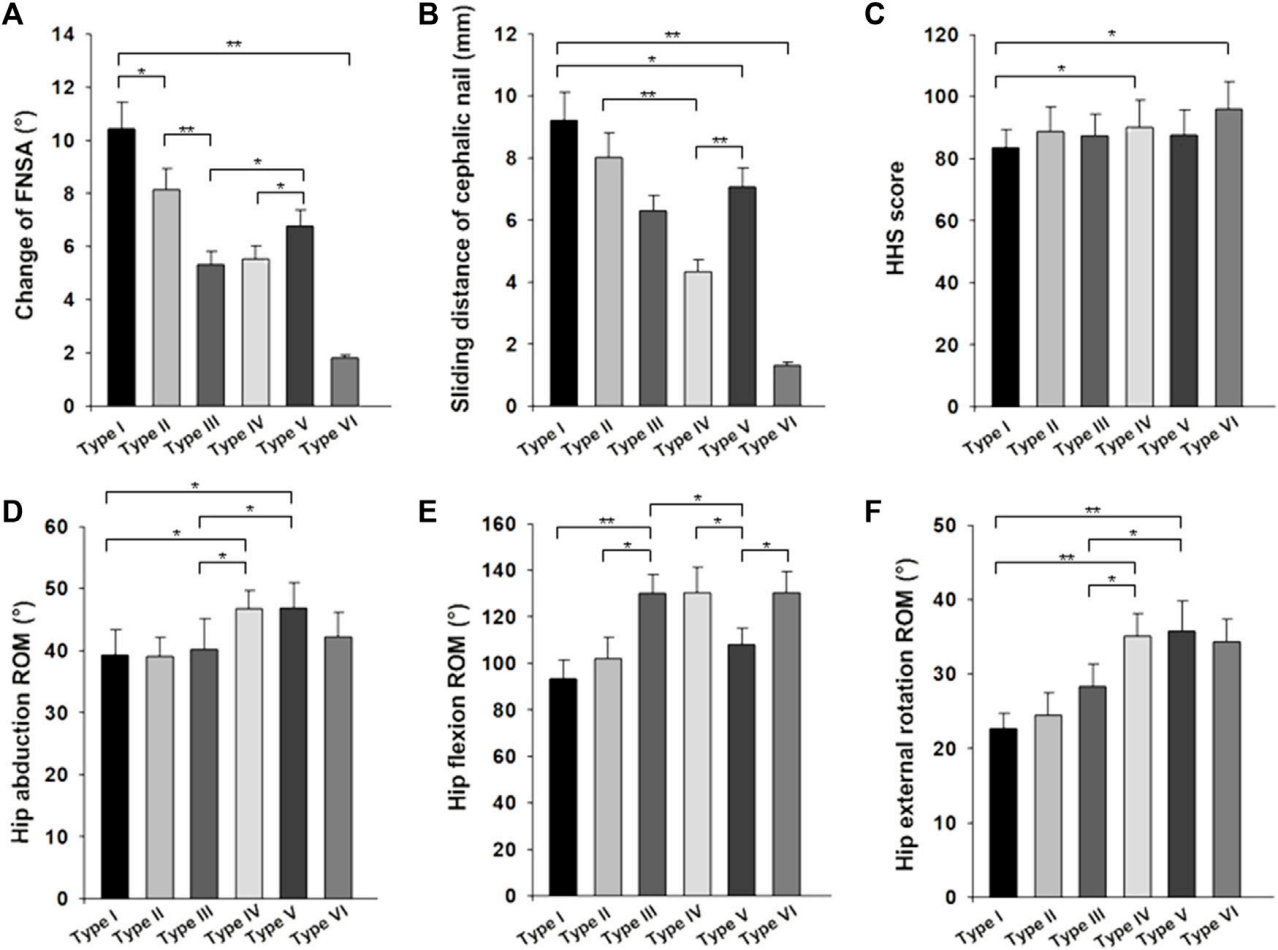
**(A)** The change of femoral neck–shaft angle (FNSA) by patient type. **(B)** The Sliding distance of cephalic nail by patient type. **(C)** The HHS score by patient type. **(D)** The hip abduction ROM by patient type. **(E)** The hip flexion ROM by patient type. **(F)** The hip external rotation ROM by patient type (*p < 0.01, **p < 0.005).

In analyzing radiography parameters, we measured the patient’s sliding distance of cephalic nail as well as femoral neck–shaft angle (FNSA) changes. The imaging results of patients in different groups are shown in Table 2 and Figure 7. We found that the sliding distance and the change of FNSA of patients in the Type I group and the Type II group were significantly different from those of other groups, while the sliding distance and the change of FNSA in the Type VI group were significantly smaller. In addition, we found a significant difference in the change in FNSA between the Type I group and the Type II group.

**TABLE 2:** Result of the change in radiography parameters, function, and range of motion for patients.

Description	Classification						
	I	II	III	IV	V	VI	*p* value
TAD (mm)	20.45 ± 5.42	18.37 ± 5.77	17.81 ± 4.51	19.57 ± 2.97	17.34 ± 3.72	18.11 ± 3.06	0.251
Change of FNSA (°)	10.44 ± 6.24	8.13 ± 4.16	5.31 ± 2.97	5.54 ± 3.04	6.77 ± 3.90	1.82 ± 1.51	0.007*
Sliding distance of cephalic nail (mm)	9.22 ± 5.24	8.01 ± 4.71	6.30 ± 3.47	4.33 ± 2.92	7.07 ± 2.79	1.32 ± 1.23	0.001*
HHS score	85.54 ± 6.34	88.72 ± 7.10	87.34 ± 4.79	90.01 ± 5.04	87.74 ± 4.81	95.52 ± 1.24	0.009*
Hip abduction ROM (°)	39.31 ± 15.21	39.11 ± 12.07	40.21 ± 7.60	46.72 ± 4.46	46.91 ± 3.22	42.21 ± 13.57	0.003*
Hip adduction ROM (°)	19.52 ± 13.05	22.81 ± 12.41	27.34 ± 8.78	24.24 ± 7.90	21.86 ± 10.45	23.33 ± 7.87	0.233
Hip flexion ROM (°)	93.41 ± 28.20	102.10 ± 25.82	130.22 ± 23.78	130.35 ± 24.05	108.17 ± 20.92	130.37 ± 14.44	0.002*
Hip posterior extension ROM (°)	20.11 ± 12.54	21.81 ± 14.34	27.34 ± 9.87	25.24 ± 8.00	24.86 ± 6.07	26.33 ± 10.24	0.162
Hip internal rotation ROM (°)	28.34 ± 11.80	27.65 ± 13.48	34.67 ± 12.33	35.97 ± 8.74	30.86 ± 8.41	37.85 ± 7.64	0.327
Hip external rotation ROM (°)	22.72 ± 12.44	24.50 ± 11.73	28.34 ± 13.21	35.07 ± 7.89	35.78 ± 8.91	34.33 ± 8.32	0.007*

TAD, tip–apex distance; FNSA, femoral neck–shaft angle.

*p < 0.05 was considered significant.

The function and range of motion of different groups are presented in Table 2 and Figure 7. The HHS score of the Type I group was 85.54 ± 6.34, significantly lower than that of the other five groups, there was a significant difference between the six groups, but only statistically different between type IV and VI (*p* < 0.01).

The range of motion (external rotation and abduction of the hip joint) in patients with type I and II fractures was significantly less than that in the other three groups, there was a significant difference between the six groups. Moreover, the differences between type I and type II patients and the VI and V groups were statistically significant. The range of motion (external rotation and abduction) of the Type VI group was less than that in the other two groups, but it was not statistically significance. There is a significant difference in postoperative flexion activity between all the groups, with patients in the Type I, Type VI, and Type V groups was significantly less than that of the other three groups, and the difference was statistically significant.

## Discussion

In this study, we retrospectively modeled 348 intertrochanteric fractures imaged by CT scan, and applied 3-D fracture-mapping techniques to macroscopically analyze the fragment morphology of medial, posteromedial, and posterolateral fractures (Xie et al., 2017). We divided posterior-medial fractures into six different classifications for comparison. Our classification system differs from previous classification systems in that this study more accurately presents the fracture line and fragment morphology of posterior-medial fractures through an advanced fracture-mapping technique, which allows us a more accurate understanding of posterior-medial injuries. In posterior-medial injuries, whether medial, posteromedial, or posterolateral, there are different injury patterns, and the resulting postoperative effects are distinct. By analyzing the posterior-medial fracture pattern, we can further improve our understanding of this fracture type.

In recent years, the popularity of 3-D methods for analysis by CT has also grown. In 2017, Shoda et al. (2017) proposed the classification of intertrochanteric fractures by 3D-CT. In 2019, Li et al. (2019) further classified femoral fractures into five classifications using the Hausdorff distance-based K-means approach (Li et al., 2019). However, we believe that a comprehensive fracture classification should not only accurately diagnose fractures, guide treatment, and predict the prognosis of fractures, but also be reproducible and simple for use by clinicians across the scope of practice.

In our study and classification, we did not describe the lateral wall, because we believe that intertrochanteric fractures involving the lateral wall are a special type of femoral fracture, and their fracture patterns and prognosis are considerably different from other intertrochanteric fracture models. In addition, we believe that the posteromedial structure plays an important biomechanical role in ensuring the stability of the proximal femur (Sharma et al., 2014; Ciufo et al., 2017; Sharma et al., 2017). The loss of support in the posteromedial side is an important cause of femoral head collapse, femoral neck shortening, and internal fixation failure, and studies of the posterior-medial fracture line and fracture fragments have already been reported. In 2017, Sharma et al. (2017) described the size, shape, and fracture mode of the smaller trochanteric fragments. Xiong et al. (2019) further summarized the extended fragment of the lesser trochanter and posterior cortex. Their study provided further insight into the characteristics of posterior-medial fracture structures, but further analyses of the clinical morphology of different fragment morphologies are lacking. Our study classification targets only the posterior-medial fragment of intertrochanteric fractures, and we believe that the inferential pattern of fracture-mapping technique gives us a new spatial perspective on the fracture pattern and morphology of the posterior-medial fragments after intertrochanteric fractures, and will ultimately allow us to link fracture morphology to clinical features.

In our study, posterior-medial intertrochanteric fractures were typed into six classifications. Both type I and type II fracture lines involve the entire posterior-medial intertrochanteric region, leaving the posterior-medial cortex unsupported and the fracture extremely unstable. Moreover, the sliding distance of cephalic nail and FNSA change in the radiographic findings of type I and type II were significantly different from those of the other groups (Table 2; Figure 7). However, even if the extent of type I and type II fracture involvement is consistent, the clinical results are different due to the uniqueness of intertrochanteric fractures. The concept of the “isolated fragment” that we introduced divides intertrochanteric fractures involving the entire posterior-medial fragment into two classifications. We can find in the fracture-mapping technique that the fracture of type I is more complex and is a posterior-medial comminuted fracture. Type II fractures, on the other hand, are relatively simple, and most of the posteromedial aspect remains a complete fragment. There is also a significant difference between type I and type II in the radiography parameters of FNSA change (Figure 7). We believe that type I fractures have a worse outcome than type II fractures and more surgical complications, but further studies are needed to confirm this hypothesis. Although the type IV fracture shown in Figure 7 involves the medial wall fracture, there is no significant difference in the imaging indicators. We trust that the posterior wall will play a partial supporting role in the case of medial wall fracture. At this time, the fracture is still stable when the calcar femorale is intact. As expected, we found that the radiographic appearance of type VI fractures was significantly different from that of the other classifications, further confirming the important role of the posterior-medial fragment in maintaining the stability of intertrochanteric fractures. However, the stability of intertrochanteric fractures involving medial wall injury was not universally affected; medial wall injury accounted for (97.4%) of the patients with posterior-medial fractures, but the radiographic results in patients with type III, IV, and V fractures suggested that the fracture type was relatively stable. HHS function scores indicated a significant difference between type I fracture and other fracture classifications, which also confirmed that neck shortening significantly reduced Harris hip scores (Weil et al., 2012; Slobogean et al., 2017; Felton et al., 2019; Shin et al., 2020). Moreover, the postoperative HHS function score of type II fracture with the same posterior-medial involvement was satisfactory.

We (and many researchers) believe that the entire pathophysiological process of fracture is related to muscle and ligament attachment at the fracture site (Bair and Zafar Gondal, 2020; Slagstad et al., 2020). This belief guided our classification of posterior-medial fragment into posterolateral, posteromedial, and medial groups due to muscle attachment in the intertrochanteric region and its different roles between the trochanters (Marmor et al., 2013; Ehrnthaller et al., 2017; Ren et al., 2019), where both posteromedial and posterolateral are muscle attachments and the medial region is an important supporting structure. Through fracture-mapping, we found that the direction of the fracture line was consistent with this mechanistic view, confirming the reliability and accuracy of our classification. In addition, we also considered the piriformis, obturator internus, obturator externus, superior gemellus, inferior gemellus, and the quadratus to be the external rotator muscle groups with the end points at the piriformis fossa and intertrochanteric ridge of the femur. The gluteus medius and gluteus minimus were the abductor muscle groups with end point at the greater trochanter, while the iliac muscle and psoas were the flexor muscle groups with end points at the lesser trochanter. Therefore, we retrospectively analyzed the postoperative hip range of motion of patients and found that different fracture classifications had unique postoperative range of motion effects, and even the posterolateral fracture alone had a significant effect on hip abduction function. This finding can further guide individualized postoperative functional rehabilitation of patients using hip joint exercise.

This study has several limitations. First, our study is a retrospective study in which we reviewed patients managed operatively for intertrochanteric fractures, while most patients with type VI fractures were managed conservatively and not included in our analysis. As a result, we had a lower proportion of type VI fractures in our study. Second, some patients with type I fractures could not be systematically classified because the fracture was severely displaced and separated into very small fragments. In such patients, fracture morphology of type I is less complex than that of type I fractures, which is not captured by fracture-mapping technique. Finally, the isolated posteromedial + posteromedial group was not represented in our study population. This may be related to our insufficient number of patients, or other yet undefined mechanisms that need to be further elucidated.

## Conclusion

In conclusion, the results of this morphologic study helps to further identify and recognize the characteristics of posterior-medial intertrochanteric fracture patterns, which may be strongly associated with different clinical outcomes. More prospective randomized controlled trials are needed to verify the effectiveness of this new classification system.

## Data Availability

The original contributions presented in the study are included in the article/Supplementary Material, further inquiries can be directed to the corresponding authors.

## Abbreviations

3-D, three-dimensional; FNSA, femoral neck–shaft angle; TAD, tip–apex distance; HHS, hip harris score.
